# Optimization of target biochar for the adsorption of target heavy metal ion

**DOI:** 10.1038/s41598-022-17901-w

**Published:** 2022-08-11

**Authors:** Runjuan Zhou, Ming Zhang, Shuai Shao

**Affiliations:** School of Architecture and Civil Engineering, Anhui Polytechnic University, 8 Middle Beijing Road, Wuhu, 241000 Anhui People’s Republic of China

**Keywords:** Environmental sciences, Natural hazards

## Abstract

The purpose of this work is to study the pyrolysis conditions of target biochar suitable for target heavy metal ion, to characterize the optimized target biochar, and to study the adsorption performance of biochar. With Cu^2+^ and Zn^2+^ as the target pollutants, the pyrolysis conditions involved in the preparation process as pyrolysis temperature, pyrolysis time, and heating rate were evaluated and optimized from Box–Behnken Design (BBD), response surface methodology (RSM) and desirability function, the optimized pyrolysis conditions of target biochar for Cu^2+^ (Cu-BC) and Zn^2+^ (Zn-BC) were obtained. The optimum pyrolysis parameters for Cu-BC and Zn-BC were pyrolysis time of 3.09 and 2.19 h, pyrolysis temperature of 425.27 and 421.97 °C, and heating rate of 19.65 and 15.88 °C/min. The pseudo-second-order kinetic and Langmuir isotherm model proved to be the best fit for the equilibrium data, with a maximum adsorption capacity (*Q*_*max*_) fitted by Langmuir model were 210.56 mg/g for Cu^2+^ by Cu-BC and 223.32 mg/g for Zn^2+^ by Zn-BC, which were both higher than the *Q*_*max*_ of unoptimized biochar (BC) for Cu^2+^ (177.66 mg/g) and Zn^2+^ (146.14 mg/g). The physical properties, chemical structure, surface chemistry properties of Cu-BC and Zn-BC were characterized by Zeta potential meter, Scanning electron microscopy with energy dispersive X-ray spectroscopy (SEM-EDX), Fourier-transform infrared spectroscopy (FTIR), and X-ray diffraction (XRD). This study puts forward a new perspective for optimizing target biochar production for special environmental application.

## Introduction

Biochar, a kind of stable carbon rich material with high level aromatization generated by pyrolysis of biomass under oxygen-limited conditions^1,2^. Biochar has been widely used in the remediation of heavy metal ions in water bodies due to its unique pore structure, large specific surface area and complex surfacial-active functional groups, as well as its great potential in the adsorption and removal of heavy metals^3,4^. However, in practical application, the adsorption performance of biochar for heavy metal ions is affected by many factors, such as biomass species, preparation conditions, pH, dosage of biochar, react time, types and concentration of heavy metal ion, etc.^3^. Therefore, the study on the factors affecting the adsorption of heavy metals by biochar is beneficial to improve the adsorption effect in practical application.

Although there are many factors affecting the adsorption performance of biochar, for fixed biomass and fixed heavy metal ions, the preparation conditions become one of the most important factors. The preparation conditions of biochar mainly include pyrolysis temperature, pyrolysis time and heating rate, among these conditions the pyrolysis temperature has a significant effect on the performance of biochar^5–7^. Pyrolysis temperature has an effect on the elemental composition, cation exchange capacity, surface oxygen-containing functional groups, aromization degree, specific surface area, pore structure and alkalinity of biochar^6,8^. Studies have shown that with the increase of pyrolysis temperature, the content of hydrogen, sulfur, nitrogen and other elements in biochar and the number of oxygen-containing surface functional groups decrease, the cation exchange capacity decreases, and the degree of aromatization increases. These changes are not favorable for the adsorption of biochar to heavy metal ions. As the temperature increases, the specific surface area, pore structure and alkalinity of biochar increase, which is conducive to the adsorption of heavy metal ions^7–11^. Pyrolysis time mainly affects the composition, specific surface area and pore structure of biochar, while heating rate mainly affects the yield of biochar^12–14^. These properties of biochar have an impact on the adsorption performance of biochar, but these properties need to be characterized by the corresponding instrument.The traditional biochar production process is: pyrolysis, then characterization, and finally application. The role of characterization is mainly used to evaluate the performance of biochar. At present, the evaluation of biochar adsorption performance mainly focuses on functional groups, surface structure, porosity, specific surface area, and so on^15,16^. Although these characteristics are good for evaluating the adsorption performance of biochar, the analysis of these characteristics requires advanced equipment. If biochars produced under every preparation conditions are characterized, the cost of analysis is too high and it is not economically feasible. Therefore, it is necessary to find some indicators that are easy to operate, less characterization times, low cost, and can directly reflect the adsorption capacity of biochar on pollutants to judge the adsorption performance. In the application of biochar to adsorb heavy metal ions, biochar's function is to adsorb and remove heavy metal ions. Therefore, if a specific heavy metal ion is taken as the target pollutant, the adsorption capacity of biochar to the target pollutant is taken as the index to investigate the preparation conditions of biochar. We only characterize the biochar with the strongest adsorption capacity, which can not only reduce the number of characterized samples, reduce the cost, but also more intuitively reflect the biochar's adsorption capacity of target pollutants, and finally obtain the biochar with the best adsorption capacity of this kind of heavy metal ions. Different heavy metal ions have different properties. Therefore, for a certain biochar, its adsorption performance for different heavy metal ions is different. For the same biochar, although it can adsorb heavy metal ions in water, its adsorption performance of different heavy metal ions is different, so the adsorption capacity of some heavy metal ions is limited and the adsorption selectivity is poor^4^. Therefore, we proposed the concept of target biochar for target heavy metal ion. In the authors' previous study, the optimal preparation conditions of biochar were investigated according to the adsorption capacity of Cd^2+^ and Pb^2+^, and the studies found that different heavy metal ions corresponded to different preparation conditions of biochars, which we called the target biochar^17,18^. In order to further prove the correctness of this view,, this research took Cu^2+^ and Zn^2+^ as target pollutants, water hyacinth as biomass material for biochar, and adopted response surface methodologies (RSM) to optimize the preparation conditions (pyrolysis temperature, pyrolysis time and heating rate) that affect the adsorption performance of biochar, and the target biochar BC-Cu (biochar for Cu^2+^) and BC-Zn (biochar for Zn^2+^) were obtained, respectively. Only BC-Cu and BC-Zn need to be characterized, which greatly reduced the number of characterization samples and the production cost. The adsorption kinetics and isotherms of target biochars for target heavy metal ions were studied. This study provides theoretical and technical support for the preparation of the target biochar to remove the target pollutants. The proposed method of removing target heavy metal ions with target biochar can not only save biomass, time and cost for the production of biochar, but also only characterize the target biochar to reduce the number of characterization, thus reducing the characterization cost. More importantly, in terms of the final removal effect, the target heavy metal ions correspond to the target biochar, which has a better removal effect compared with the traditional biochar preparation method. Therefore, this study provides theoretical guidance and technical support for "precise control of pollutants" of biochar.

## Materials and methods

### Materials

The water hyacinth in this study was collected from the river inside the Anhui Polytechnic University in Wuhu, Anhui Province, China.

All chemical reagents had analytical reagent grade and were purchased from Aladdin Reagent (Shanghai) Co., LTD, which includes Cu(NO_3_)_2_·3H_2_O, Zn(NO_3_)_2_·6H_2_O, sodium hydroxide (NaOH) and nitric acid (HNO_3_). In this study, all experiments were performed using ultrapure water.

### Preparation of biochar

Cut the airing water hyacinth into 1–2 cm segments, and dried them in drying oven at 70 °C until constant weight. The dried water hyacinth was put into the high temperature resistant quartz boat, and then placed in a tubular furnace. The preparation method is shown in the previous study^17,18^. The pyrolysis parameters (pyrolysis temperature, pyrolysis time and heating rate) were set according to the experimental requirements, and the whole process was carried out under the protection of nitrogen. The specific experimental operations of the three preparation parameters are as follows:

1) Pyrolysis temperature.

The pyrolysis time and heating rate were fixed at 2 h and 20 °C/min, respectively. The pyrolysis temperature was selected as 200, 300, 400, 500, 600 and 700 °C, respectively.

2) Pyrolysis time.

The pyrolysis temperature and heating rate were fixed at 400 °C and 20 °C/min, respectively. The pyrolysis time was selected as 1, 2, 3, 4 and 5 h, respectively.

3) Heating rate.

The pyrolysis temperature and pyrolysis time were fixed at 400 °C and 2 h, respectively. The heating rate was selected as 5, 10, 15, 20, 25 and 30 °C/min, respectively.

### Adsorption experiments of target biochars

1. Screening of central value of preparation conditions for target biochar.
  In order to investigate the central value of preparation conditions on the adsorption of target pollutants by biochar, 2.0 g/L of biochars were reacted with 20 mg/L of Cu^2+^ or Zn^2+^ solutions for 240 min on the oscillator SHA-CA at 150 rpm/min at 25 °C. The Cu^2+^ and Zn^2+^ stock solutions were prepared with Cu(NO_3_)_2_·3H_2_O and Zn(NO_3_)_2_·6H_2_O, respectively.
  The adsorption capacity (*q*_*t*_) of heavy metal ion was calculated by Eq. (1)^17,18^:
  1 $${q}_{t}=\frac{\left({C}_{0}-{C}_{t}\right)V}{m}$$
  *C*_*0*_: the initial concentration of Cu^2+^ or Zn^2+^ (mg/L); *C*_*t*_: the concentration of Cu^2+^ or Zn^2+^ at *t* time (mg/L); *m*: the weight of biochar (g); *v*: the volume of Cu^2+^ or Zn^2+^ solution (L).
2. Optimization of preparation conditions for target biochar.
  With Cu^2+^ or Zn^2+^ adsorption capacity of biochar as response, the preparation conditions of the target biochar were optimized by using the Box–Behnken Design (BBD), and the interaction between various conditions were explored by response surface methodology (RSM). As the operation conditions, the pyrolysis time (*X*_*1*_), pyrolysis temperature (*X*_*2*_), and heating time (*X*_*3*_) were investigated by RSM, and the Cu^2+^ or Zn^2+^ adsorption capacity of biochars was selected as the response variable (*Y*_*Cu*_ or *Y*_*Zn*_). The experimental results were analyzed and fitted to a quadratice quation by using the Design Expert 10.0 software. In the optimization experiment, the concentration of heavy metal ions changed to 50 mg/L, and other experimental conditions were same as the preliminary test. The biochars prepared under optimized preparation conditions were called Cu-BC (for Cu^2+^) and Zn-BC (for Zn^2+^).
3. Adsorption kinetics of target biochar for target heavy metal ions.
  The adsorption kinetics of target biochars for the heavy metal ions were determined by dosing 1.0 g of biochar in 1000 mL of solutions with 50 mg/L Cu^2+^ or Zn^2+^, and the react time was 0, 30, 60, 90, 120, 150, 180, and 240 min. The fitting models are shown in Eqs. (2) and (3)^17,18^.
  Pseudo-first-order model:
  2 $${q}_{t}={q}_{e}\left(1-exp\left(-{k}_{1}t\right)\right)$$
  Pseudo-second-order model:
  3 $${q}_{t}=\frac{{q}_{e}^{2}{k}_{2}t}{1+{q}_{e}{k}_{2}t}$$
  *q*_*e*_ and *q*_*t*_ are adsorption capacity of Cu^2+^ or Zn^2+^ adsorbed by biochar at equilibrium and *t* time, respectively (mg/g); *k*_*1*_: the pseudo-first-order rate constant (1/min); *k*_*2*_: the pseudo-second-order rate constant (g/mg min).
4. Adsorption isotherm experiments of target biochar for target heavy metal ions.
  Adsorption isotherm experiments for target heavy metal ions were conducted by dosing 0.2 g of target biochars in 100 mL of solutions with a range of Cu^2+^ and Zn^2+^ concentrations (10, 20, 50, 100, 200, 500, 800 and 1000 mg/L).
  The pH values of the heavy metal solutions in all batch experiments were adjusted to about 5.5 by 0.1 mol/L HNO_3_ and NaOH solutions. The concentrations of Cu^2+^ and Zn^2+^ after adsorption were determined by Shimazu ICPE-9000 inductively coupled plasma emission spectrometer. All experiments were repeated in triplicate. The equilibrium data were analyzed by the Langmuir and Freundlich models, and the two models were shown in Eqs. (4) and (5)^17,18^.
  Langmuir model:
  4 $${q}_{e}=\frac{{K}_{a}{Q}_{m}{C}_{e}}{1+{K}_{a}{C}_{e}}$$
  Freundlich model:
  5 $${Q}_{e}={K}_{F}{C}_{e}^\frac{1}{n}$$
  *q*_*e*_ or *Q*_*e*_: adsorption capacity of Cu^2+^ or Zn^2+^ by biochar at equilibrium (mg/g); *C*_*e*_: concentration of Cu^2+^ or Zn^2+^ at equilibrium (mg/L); *Q*_*m*_: the Langmuir maximum adsorption capacity (mg/g); *K*_*a*_: Langmuir constant (L/mg); *K*_*F*_: the Freundlich constant; 1/n: adsorption strength of the system.

### Characterization of target biochars

The characterization methods of biochar were detailed in previous research^17,18^. Scanning electron microscope microscopy and energy dispersive spectroscopy (SEM–EDX) analysis was conducted using S-4800 scanning microscope and energy dispersive X-ray spectroscopy (Hitachi, Japan) to examine the surface topography and the element types and contents of biochar. Specific surface area of biochar was determined using a NOVA 2000e analyzer. Nitrogen adsorption–desorption was carried out in 77 K liquid nitrogen. The specific surface area and pore size distribution of the adsorbed material were calculated according to the peak area of adsorption and desorption. The functional groups of biochars were analyzed by an SHIMADZU IRPrestige-21 transform infrared spectrometer (Shimadzu, Japan). 1 mg sample powder was mixed with KBr at the mass of 1:200 and ground evenly. After pressing, the samples were put into the sample chamber and scanned in the wavelength range of 400-4000/cm. The crystallinity changes of the three materials were characterized by X-ray diffraction (XRD) with Bruck-D8 series X-ray (powder) diffractometer (Bruck, German). 0.2 g of sample powder was taken and put into the sample chamber after tablet pressing, and scanned in the range of 10°–80°.

### Analysis methods

The data and figures were analyzed by OriginPro 2017 (OriginLab Corporation, Northampton, MA, USA). The optimize experiments data and figures were designed by the soft of Design-Expert 10.0 (Stat-Ease. Inc., Minneapoils, MN, USA).

## Results and discussions

### Preliminary test: determination of minimum, intermediate and maximum ranges

**Figure 1:**
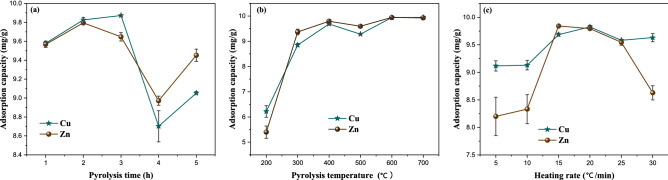
Effects of pyrolysis conditions on the adsorption capacity of Cu^2+^ and Zn^2+^ by BC.

Adsorption capacity of Cu^2+^ and Zn^2+^ of biochars under various production conditions were shown in Fig. 1a–c.

Figure 1a showed the pyrolysis time effects on the adsorption capacity of Cu^2+^ and Zn^2+^ by biochars. It can be seen that the adsorption capacity of Cu^2+^ and Zn^2+^ by biochars increased first and then decreased with the pyrolysis time prolonging. According to the Fig. 1a, the optimal pyrolysis time of biochar corresponding to Cu-adsorbed biochar is 3 h, and the maximum adsorption capacity of Cu^2+^ is 9.87 mg/g, while the optimal pyrolysis time of biochar corresponding to Zn-adsorbed biochar is 2 h, and the maximum adsorption capacity of Zn^2+^ is 9.79 mg/g. According to the above experimental results, the pyrolysis time of 3 h and 2 h were selected as the center values for Cu-adsorbed and Zn-adsorbed biochar, respectively. So, in the optimization experiments, the low, medium and high levels for Cu-adsorbed biochar were 2 h (− 1), 3 h (0) and 4 h (+ 1), and for Zn-adsorbed biochar were 1 h (− 1), 2 h (0) and 3 h (+ 1). The codes − 1, 0, and + 1 indicate low, medium and high levels of preparation parameters.

The effect of pyrolysis temperature on the adsorption property of biochars is displayed in Fig. 1b. From the Fig. 1b, the adsorption capacity of biochars on Cu^2+^ and Zn^2+^ improved with the increase of pyrolysis temperature. When the pyrolysis temperature increased to 400 °C, the adsorption capacity of biochar for Cu^2+^ and Zn^2+^ reached 9.68 mg/g and 9.79 mg/g, respectively. Continued to increase the pyrolysis temperature to 500 °C, the adsorption capacity decreased slightly, while the pyrolysis temperature is further increased to 600 °C and 700 °C, the adsorption capacity of Cu^2+^ and Zn^2+^ were higher than that of 500 °C, but lower than that of 400 °C. Therefore, 400 °C was chosen as the optimal temperature for the production of biochar from water hyacinth adsorbing Cu^2+^ and Zn^2+^, and the three levels of pyrolysis temperature in BBD were 300 °C (− 1), 400 °C (0) and 500 °C (+ 1).

Figure 1c represented the effect of heating rate on the adsorption capacity of biochars for Cu^2+^ and Zn^2+^. With the increase of heating rate, the lignocellulosic structure of water hyacinth gradually decomposed, and the volatiles precipitated promoted the formation of biochar pores. When the heating rate continues to increase, the volatiles escape sharply, the residual pores become larger or some products block the pores, as a result, the specific surface area of the prepared biochar decreased, and the adsorption performance of the biochar is reduced. From the Fig. 1c, when the heating rate was 20 °C/min, the adsorption capacity of biochar on Cu^2+^ was the highest, reaching 9.83 mg/g, while for Zn^2+^, the optimal heating rate was 15 °C/min, the adsorption capacity of Zn^2+^ was 9.84 mg/g. Therefore, 20 °C/min and 15 °C/min were selected as the center values for preparing biochar to adsorb Cu^2+^ and Zn^2+^ in the BBD, respectively. The three levels of heating rate in the BBD were 15 °C/min (− 1), 20 °C/min (0) and 30 °C/min (+ 1) for Cu-adsorbed biochar, and 10 °C/min (− 1), 15 °C/min (0) and 20 °C/min (+ 1) for Zn-adsorbed biochar.

### Optimal preparation conditions of target biochars

Table 1 summarized the actual and coded values of the preparation parameters for biochars. Meanwhile, a total of 17 runs of randomized BBD experiments were designed, and the corresponding adsorption capacities of Cu^2+^ and Zn^2+^ are listed in Table 2.

**Table 1:** Coded and actual values for independent variables.

Target ions	Variables	Symbol	Coded values		
			− 1	0	1
Cu^2+^	Pyrolysis time (h)	*X*_*1*_	2	3	4
	Pyrolysis temperature (°C)	*X*_*2*_	300	400	500
	Heating rate (°C/min)	*X*_*3*_	15	20	25
Zn^2+^	Pyrolysis time (h)	*X*_*1*_	1	2	3
	Pyrolysis temperature (°C)	*X*_*2*_	300	400	500
	Heating rate (°C/min)	*X*_*3*_	10	15	20

**Table 2:** BBD experimental runs for the optimization of preparation parameters for Cu-BC and Zn-BC.

Run	*X*_*1*_	*X*_*2*_	*X*_*3*_	Adsorption capacity (mg/g)	
				Cu^2+^	Zn^2+^
1	0	1	1	16.07	17.03
2	− 1	0	− 1	15.75	16.56
3	1	0	− 1	16.14	17.83
4	0	0	0	17.83	19.14
5	1	1	0	16.23	17.13
6	1	− 1	0	13.73	14.16
7	− 1	1	0	15.57	16.94
8	− 1	− 1	0	15.04	13.84
9	0	1	− 1	15.86	16.09
10	0	0	0	17.72	19.61
11	− 1	0	1	14.49	17.19
12	0	− 1	1	14.23	14.54
13	0	0	0	18.17	19.64
14	0	0	0	18.20	19.49
15	0	0	0	18.12	19.29
16	1	0	1	15.69	18.51
17	0	− 1	− 1	14.53	14.68

The response model was obtained by fitting the experimental results with quadratic multiple regression equation. The response surface methodology (RSM) was used to construct the response relationship between *Y*_*q(Cu)*_, *Y*_*q(Zn)*_ (*Y*_*q(Cu)*_ and *Y*_*q(Zn)*_ representing the adsorption capacity of biochar for Cu^2+^ and Zn^2+^ respectively) and preparation conditions (*X*_*1*_, *X*_*2*_ and *X*_*3*_ represent pyrolysis time, pyrolysis temperature and heating rate, respectively), and the interaction between preparation conditions and their influence on adsorption capacity were studied. The response models are shown in Eqs. (6) and (7), and in the model, the positive sign (+) represent the synergistic effect and the negative sign (−) indicates antagonistic effect^19^.

6 $${Y}_{q(Cu)}=18.01+0.12{X}_{1}+0.77{X}_{2}-0.22{X}_{3}+0.49{X}_{1}{X}_{2}+0.2{X}_{1}{X}_{3}+0.13{X}_{2}{X}_{3}-1.26{X}_{1}^{2}-1.60{X}_{2}^{2}-1.23{X}_{3}^{2}$$

7 $${Y}_{q(Zn)}=19.44+0.39{X}_{1}+1.25{X}_{2}+0.26{X}_{3}-0.033{X}_{1}{X}_{2}+0.01{X}_{1}{X}_{3}+0.27{X}_{2}{X}_{3}-0.99{X}_{1}^{2}- 2.93{X}_{2}^{2}-0.92{X}_{3}^{2}$$

Analysis of variance (ANOVA) was performed on the experimental results of BBD, and the analysis results were shown in Table 3.

**Table 3:** ANOVA results for response surface quadratic models for Cu^2+^ and Zn^2+^ adsorption efficiency.

Sources	Sum of Squares	df	Mean square	*F*-value	*p*-value	Remark
**(a) Cu model**	33.17	9	3.69	22.27	0.0002	Significant
*X*_*1*_	0.11	1	0.11	0.66	0.4425	
*X*_*2*_	4.79	1	4.79	28.97	0.0010	Significant
*X*_*3*_	0.40	1	0.40	2.43	0.1631	
*X*_*1*_*X*_*2*_	0.97	1	0.97	5.87	0.0459	Significant
*X*_*1*_*X*_*3*_	0.16	1	0.16	0.97	0.3573	
*X*_*2*_*X*_*3*_	0.067	1	0.07	0.41	0.5444	
*X*_*1*_^*2*^	6.71	1	6.71	40.52	0.0004	Significant
*X*_*2*_^*2*^	10.83	1	10.83	65.43	< 0.0001	Significant
*X*_*3*_^*2*^	6.37	1	6.37	38.51	0.0004	Significant
Residual	1.16	7	0.17	–	–	
Lack of fit	0.97	3	0.32	6.81	0.0475	
Pure error	0.19	4	0.05	–	–	
*R*^2^ = 0.9662, *R*_*adj*_^2^ = 0.9229, Adequate Precision = 12.8699						
**(b) Zn model**	61.59	9	6.84	35.64	< 0.0001	Significant
*X*_*1*_	1.19	1	1.19	6.22	0.0413	Significant
*X*_*2*_	12.43	1	12.43	64.73	< 0.0001	Significant
*X*_*3*_	0.56	1	0.56	2.91	0.1320	
*X*_*1*_*X*_*2*_	4.444E − 003	1	4.444E − 003	0.02	0.8834	
*X*_*1*_*X*_*3*_	4.340E − 004	1	4.340E − 004	2.261E − 003	0.9634	
*X*_*2*_*X*_*3*_	0.29	1	0.29	1.52	0.2579	
*X*_*1*_^*2*^	4.13	1	4.13	21.54	0.0024	Significant
*X*_*2*_^*2*^	36.08	1	36.08	187.95	< 0.0001	Significant
*X*_*3*_^*2*^	3.57	1	3.57	18.61	0.0035	Significant
Residual	1.34	7	0.19	–	–	
Lack of fit	1.16	3	0.39	8.58	0.0323	
Pure error	0.18	4	0.05	–	–	
*R*^2^ = 0.9786, *R*_*adj*_^2^ = 0.9512, Adequate Precision = 16.6178						

From Table 3, it can be found that the *F*-value for individual term of *X*_*1*_, *X*_*2*_ and *X*_*3*_ are 0.66, 28.97 and 2.43 for Cu-BC, indicating that the influence of preparation conditions on Cu^2+^ adsorption performance of biochar was as follows: pyrolysis temperature (*X*_*1*_) > heating rate (*X*_*2*_) > pyrolysis time (*X*_*3*_), and 6.22, 64.73 and 2.91 for Zn-BC, and the results showed that the influence of preparation conditions on Zn^2+^ adsorption performance of biochar was as follows: pyrolysis temperature (*X*_*1*_) > pyrolysis time (*X*_*2*_) > heating rate (*X*_*3*_). As can be seen from the above results, among the three preparation parameters, pyrolysis temperature has the greatest influence on the adsorption efficiency of Cu-BC and Zn-BC. The conclusion is consistent with many research, the pyrolysis temperature is the most effective pyrolysis factor to determine the adsorption capacity of biochar^20–22^. However, the sequence of the effects of pyrolysis time and heating rate on the two biochars was different. According to the *F*-value, the order of interaction items influence on adsorption performance of Cu-BC were *X*_*1*_*X*_*2*_ (*F*-value = 5.87) > *X*_*1*_*X*_*3*_ (*F*-value = 0.97) > *X*_*2*_*X*_*3*_ (*F*-value = 0.41), and Zn-BC were *X*_*2*_*X*_*3*_ (*F*-value = 1.52) > *X*_*1*_*X*_*2*_ (*F*-value = 0.02) > *X*_*1*_*X*_*3*_ (*F*-value = 2.261E − 003)^23^. The *p*-value < 0.05 indicates the significance of terms, less than 0.01, shows that a model term was considered as extremely significant^24–26^. In this study, the *p*-value of models for adsorption capacities of Cu^2+^ and Zn^2+^ were 0.0002 and < 0.0001, and all less than 0.01, indicated that two models were extremely significant. In this case, according to the *p*-value, the significant model terms for Cu^2+^ removal are *X*_*2*_, *X*_*1*_*X*_*2*_, *X*_*1*_^*2*^, *X*_*2*_^*2*^, *X*_*3*_^*2*^, and for Zn^2+^ removal are *X*_*1*_, *X*_*2*_, *X*_*1*_^*2*^, *X*_*2*_^*2*^, *X*_*3*_^*2*^.

The coefficient *R*^*2*^ and *R*_*adj*_^*2*^ are used to further verify the validity of the model. In this study, the *R*^*2*^ values for Eqs. (7) and (8) were 0.9662 and 0.9786, respectively. It indicates that about 96.62% and 97.86% of regression models were ascribed to the preparation conditions researched. In addition, the *R*_*adj*_^*2*^ for Cu^2+^ and Zn^2+^ were 0.9229 and 0.9512, and the *R*^*2*^ and *R*_*adj*_^*2*^ of BC-Cu and BC-Zn were all above 0.92, indicating that the model has good accuracy. The Adeq. Precision (Adequate precision) is used to indicate the signal to noise ratio (SNR). The SNR > 4 is indicated that the signal is adequate, and the model can be used to guide the design space^27^. The SNR of Cu-BC and Zn-BC are 12.8699 and 16.6178, respectively, all much higher than 4, combined with the values of *R*^*2*^ and *R*_*adj*_^*2*^, it showed that the two quadratic regression models are in good agreement with the experimental results.

**Figure 2:**
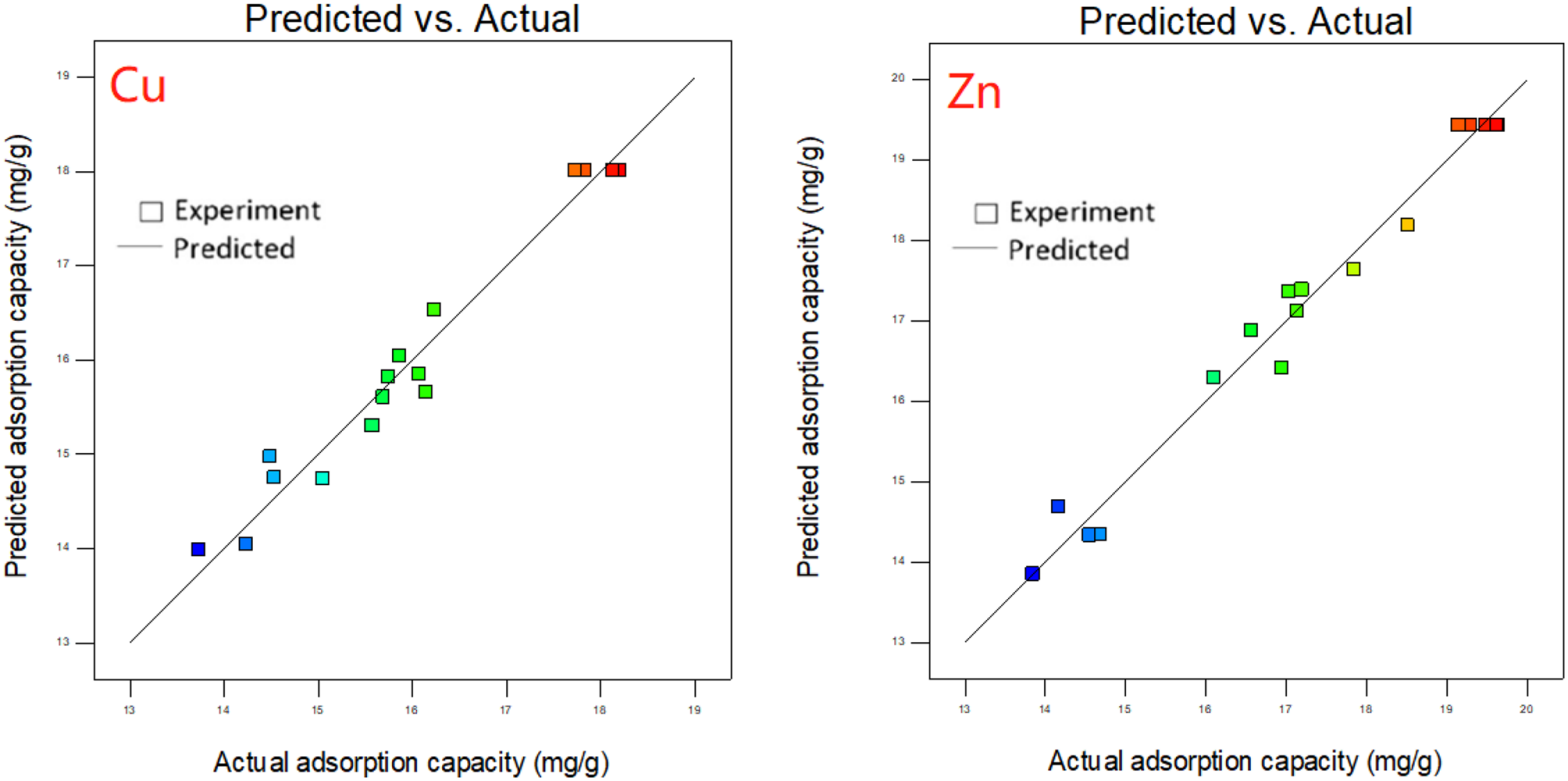
Predicted vs. experimental adsorption capacity of target biochars for target heavy metal ions.

The comparison between predicted values and actual values of adsorption capacities of Cu^2+^ and Zn^2+^ are shown in Fig. 2. As can be seen from the figure, the predicted results of the model are close to the experimental values, indicating that the correlation between the preparation conditions of target biochars and target heavy metal ions adsorption capacity.

### Effect of preparation conditions on adsorption capacities of target heavy metal ions by target biochars

**Figure 3:**
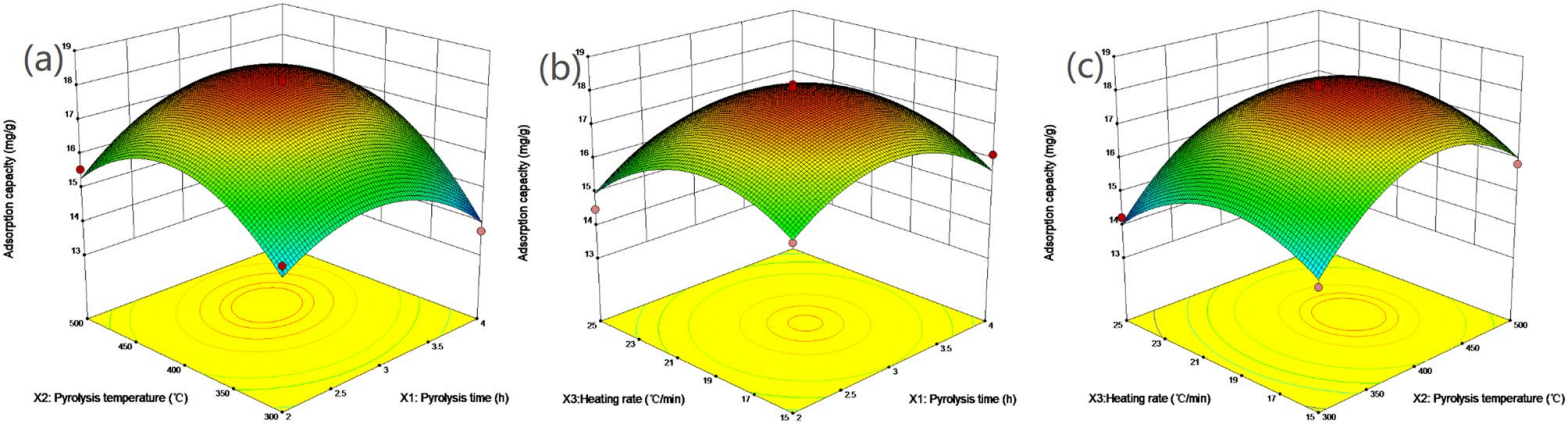
Three-dimensional (3D) plot of Cu^2+^ adsorption capacity: (**a**) influence of pyrolysis time VS pyrolysis temperature, (**b**) influence of pyrolysis time VS heating rate, and (**c**) influence of pyrolysis temperature VS heating rate (Generated by Design-Expert 10.0, Stat-Ease. Inc., Minneapoils, MN, USA, https://www.statease.com/).

**Figure 4:**
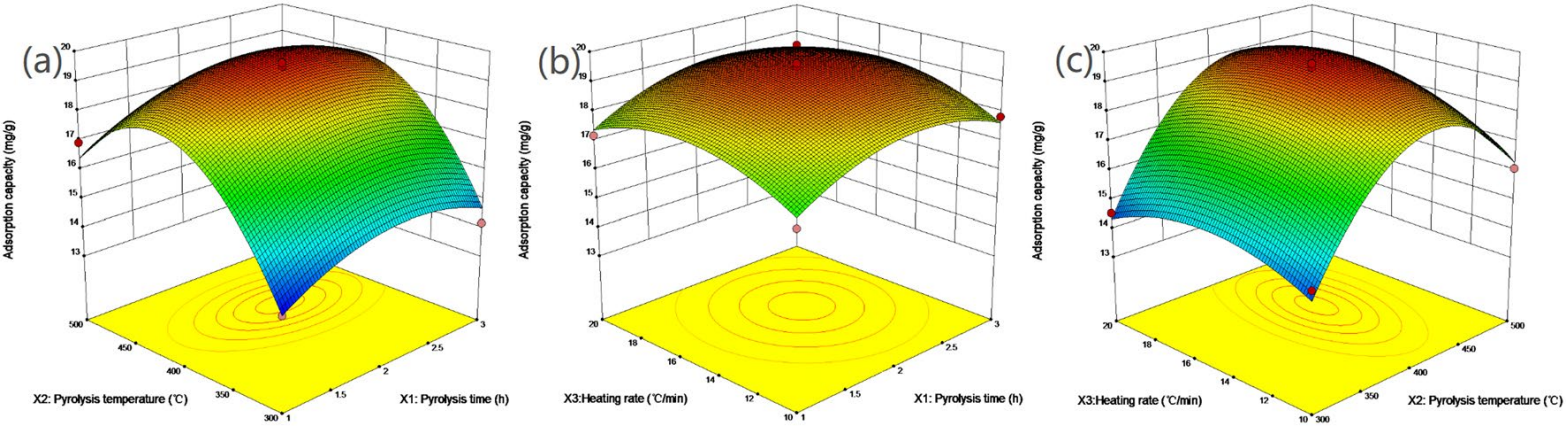
Three-dimensional (3D) plot of Zn^2+^ adsorption capacity: (**a**) influence of pyrolysis time VS pyrolysis temperature, (**b**) influence of pyrolysis time VS heating rate, and (**c**) influence of pyrolysis temperature VS heating rate (Generated by Design-Expert 10.0, Stat-Ease. Inc., Minneapoils, MN, USA, https://www.statease.com/).

According to quadratic multiple regression model Eqs. (6) and (7), the response surface three-dimensional diagram of Cu^2+^ and Zn^2+^ adsorption capacity of biochar can be obtained by the interaction of pyrolysis time (*X*_*1*_), pyrolysis temperature (*X*_*2*_) and heating rate (*X*_*3*_). In Fig. 3 (Cu) and Fig. 4 (Zn), the interaction of the pyrolysis time, pyrolysis temperature, and heating rate on the adsorption capacity of target heavy metal ions can be observed.

For Cu-BC, as shown in Fig. 3a,b, the increment of adsorption capacity of Cu-BC for Cu^2+^ occurred from 2.0 until 3.5 h of the pyrolysis time, and it reached the optimum time about 3.1 h. In terms of pyrolysis temperature, the adsorption capacity of Cu^2+^ increased from 200 to 450 °C. It can be obtained in Fig. 3a,c, the optimum pyrolysis temperature at about 400–500 °C, the adsorption capacity of Cu^2+^ by Cu-BC was increased, and the best pyrolysis temperature about 430 °C for Cu-BC. Meanwhile, Fig. 3b,c showed the heating rate for preparing Cu-BC, and the optimum heating rate is 19.5 °C/min for Cu-BC.

In Fig. 4a–c, the optimum preparation conditions for Zn-BC were shown. As for pyrolysis time, the adsorption capacity of Zn^2+^ increased gradually from 1.5 to 2.5 h, and the pyrolysis time was about 2.2 h when the maximum adsorption capacity was reached. The optimum pyrolysis temperature for Zn-BC was displayed in Fig. 4a,c, similar to the optimum pyrolysis temperature of Cu-BC, the optimum pyrolysis temperature of Zn-BC also occurs in the range of 400–500 °C, and the highest adsorption capacity was achieved about 420 °C. The heating rate for Zn-BC was shown in Fig. 4b,c, different from the Cu-BC, the optimum heating rate interval was 14–16 °C/min, and the best optimum heating rate for Zn-BC is 15.8 °C/min.

### Optimization of preparation conditions and verification of results

Through the optimization analysis of the experimental results by Design-Expert 10.0 software, the optimal combination of preparation conditions of two target biochars were obtained. For Cu-BC, the optimized parameters were pyrolysis time of 3.09 h, pyrolysis temperature of 425 °C and heating rate 19.65 °C/min, and as for Zn-BC, the optimized preparation conditions were the pyrolysis time of 2.19 h, the pyrolysis temperature of 422 °C and the heating rate of 15.88 °C/min. The validation study was conducted according to the optimization conditions of target biochars, each group of validation experiment was conducted in triplicate, and the validation results were averaged thrice. The results of verification experiments are shown in Table 4 and the validation results are displayed in Table 5. It can be clearly seen that the predicted results (18.12 and 19.63 mg/g for Cu-BC and Zn-BC, respectively) are in good agreement with the actual experimental results (18.92 and 19.98 mg/g for Cu-BC and Zn-BC, respectively), and the error is small. The factor named “desirability” from software were used to showed the best optimum parameters for output, the value of the “desirability” is closer to 1, the best conditions can be achieved^19,27,28^. In Table 5, the desirability values for Cu-BC and Zn-BC are 0.935 and 0.999, respectively, were closer to 1, and indicated that the best parameter conditions can be achieved. Therefore, the software has given a good prediction for pyrolysis conditions for M-BCs (M means heavy metal ions), and the target biochar prepared under the optimized preparation conditions has a strong adsorption capacity for the target heavy metal ion, which is technically and economically feasible.

**Table 4:** Model predicted values and validated experimental values.

Target ions	Response value (Y)	Predicted value	Experimental value1	Experimental value2	Experimental value3	Average experimental value
Cu^2+^	Q(mg/g)	18.12	18.49	19.01	19.26	18.92 ± 0.39
Zn^2+^		19.63	19.73	20.03	20.18	19.98 ± 0.23

**Table 5:** Model validation of predicted and experimental result.

Target ions	Optimized variables			Conditions	Respond
	Pyrolysis time (h)	Pyrolysis temperature (°C)	Heating rate (°C/min)		*Q* (mg/g)
Cu^2+^	3.09	425	19.65	Predicted values	18.12
				Experimental values	18.92
				Error	− 0.80
				Standard deviation	0.39
				Desirability	0.935
Zn^2+^	2.19	422	15.88	Predicted values	19.63
				Experimental values	19.98
				Error	− 0.35
				Standard deviation	0.23
				Desirability	0.999

### Target heavy metal ions adsorption kinetics of target biochars

**Figure 5:**
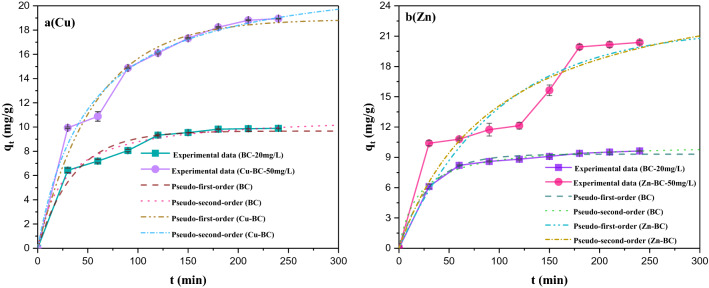
Adsorption kinetics curves (adsorption conditions: t = 25 °C, pH = 5.5, dosage = 2.0 g/L).

The kinetics curves for Cu^2+^ removal by BC and Cu-BC, and Zn^2+^ removal by BC and Zn-BC are displayed in Fig. 5a,b (BC in here is the unoptimized biochar which prepared at 2 h, 400 °C and 20 °C/min). As can be seen from the Fig. 5, the adsorption of heavy metal ions by BC and M-BCs can be divided into two stages: fast stage and slow stage. These results were consistent with the adsorption of heavy metal ions by other biochars^29–32^. This indicated that in the rapid adsorption stage, the adsorption of heavy metal ions by biochar mainly occurs on the surface of biochar, with physical adsorption predominating. After 1 h, the adsorption rate begins to slow down, and chemical adsorption controls the adsorption process until the equilibrium is reached, and in this stage, the heavy metal ions diffuse into the micropores of the biochar and bind to the inner surface of the biochar to reach the adsorption endpoint.

The BC and M-BCs showed that a similar trend displayed in kinetics, at the beginning of adsorption, the adsorption rate is fast, and about 80% of the adsorption removal occurred in first 1 h. The adsorption of BC on Cu^2+^ and Zn^2+^ reached the adsorption moderation point at about 50 min, and he adsorption equilibrium of BC for the two heavy metal ions was reached at about 120 min, while the target biochar (M-BCs) had a high adsorption rate in the first 30 min, and continued to maintain a certain adsorption level in the following time, and reached the equilibrium at about 4 h. However, in the adsorption kinetics experiment, the concentration of heavy metal ions selected was higher than that of BC due to the strong adsorption capacity of targeted biochar (M-BCs). As a result, the saturated adsorption capacity of the two biochars for heavy metal ions was greatly different, and the discussion on adsorption performance was more inclined to adsorption isotherm.

Based on the experimental results, the kinetic models were fitted, and the constants and coefficients obtained are listed in Table 6. It can be seen from Table 6, after fitting convergence, the correlation is extremely significant (*R*^*2*^ > 0.9), indicating that the adsorption of heavy metal ions by BC and M-BCs included physical adsorption and chemical adsorption. However, compared to the pseudo-first-order kinetic, as shown in Table 6, Cu^2+^ adsorption by BC and Cu-BC, Zn^2+^ adsorption by BC and Zn-BC fitted better to the pseudo-second-order kinetic (*R*_*second*_^*2*^ > *R*_*first*_^*2*^), which explained that the pseudo-second-order model preferably can well define the adsorption process of biochar for heavy metal ions. The pseudo-second-order model shows that the adsorption is mainly controlled by chemical action, not by material transport steps, and the pseudo-second-order model of reaction adsorption rate is mainly based on surface chemical reaction control, such as surface complexation and precipitation^32^.

**Table 6:** Parameters of kinetic models of Cu^2+^ and Zn^2+^.

Samples	Pseudo-first-order			Pseudo-second-order		
	*q*_*e*_ (mg/g)	*k*_*1*_	*R*^*2*^	*q*_*e*_ (mg/g)	*k*_*2*_	*R*^*2*^
BC(Cu)	9.6707	0.0277	0.8025	11.0038	0.0036	0.9172
Cu-BC	18.8935	0.0176	0.9137	23.0329	0.0008	0.9462
BC(Zn)	9.3127	0.0344	0.9468	10.3963	0.0050	0.9747
Zn-BC	21.8141	0.0102	0.7062	27.8451	0.0004	0.7490

### Target heavy metal ions adsorption equilibrium isotherms of target biochars

**Figure 6:**
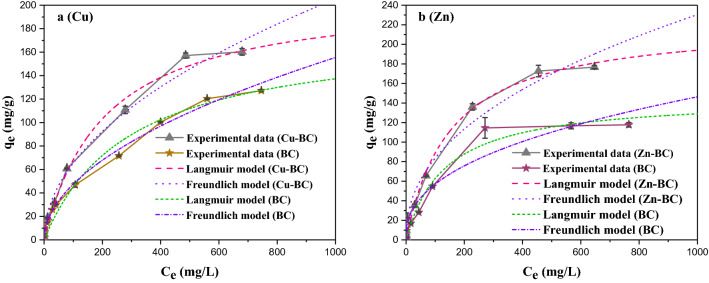
Adsorption isotherm curves of biochars.

In order to further prove that the M-BCs had better adsorption performance than that of the BC, the Langmuir and Freundlich isotherms models were used to fit the adsorption equilibrium results of BC and M-BCs for Cu^2+^ and Zn^2+^. The fitting curves and parameters of the two models are displayed in Fig. 6 and Table 7, respectively.

**Table 7:** Isotherms parameters for the adsorption of heavy metal ions.

Samples	Langmuir model			Freundlich model		
	*Q*_*max*_ (mg/g)	*K*_*a*_	*R*^*2*^	*K*_*F*_	*1/n*	*R*^*2*^
BC(Cu^2+^)	177.66	0.0034	0.9766	4.4345	0.5149	0.9926
Cu-BC	210.56	0.0048	0.9898	6.6676	0.4962	0.9894
BC(Zn^2+^)	146.14	0.0075	0.9782	3.8894	0.4799	0.9577
Zn-BC	223.32	0.0066	0.9893	10.9871	0.4405	0.9819

As can be seen from the Fig. 6a,b, the adsorption effect of Cu-BC on Cu^2+^, Zn-BC on Zn^2+^ are significantly better than that of BC. The fitting results of Langmuir model are similar to the measured values, indicating that the fitting reliability of the adsorption results is high. According to the fitting parameters in Table 7, the correlation coefficient *R*^*2*^, except for adsorption of Cu^2+^ by BC, the Langmuir model of Cu-BC, BC (Zn^2+^) and Zn-BC had a better fitting than the Freundlich model, which illustrated that a monolayer type adsorption onto the homogenous sites of biochars^16,33^. The maximum adsorption capacities (*Q*_*max*_) were fitted by the Langmuir model of BC (Cu^2+^), Cu-BC, BC (Zn^2+^) and Zn-BC were 177.66, 210.56, 146.14 and 223.32 mg/g. It was further proved that the adsorption performance of target biochars for targeted heavy metal ions was obviously stronger than that of the unoptimized biochar for heavy metal ions.

### Characterizations of biochars

**Figure 7:**
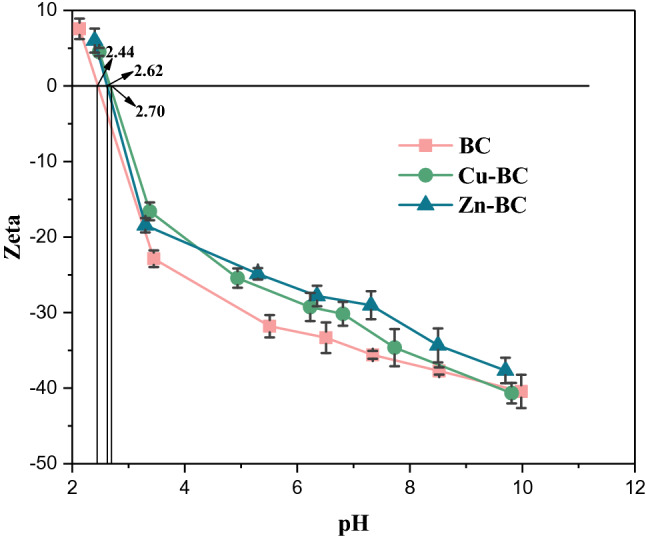
Zeta potential of BC, Cu-BC and Zn-BC.

The Zeta potential of BC, Cu-BC and Zn-BC are shown in Fig. 7. As shown in Fig. 7, the pH_pzc_ of the BC, Cu-BC and Zn-BC were 2.44, 2.70 and 2.62. When the pH value > 2.70, the BC, Cu-BC and Zn-BC all have negative charges on the surface^23^, and there will be strong electrostatic attraction between them and the positive charges on the surface of heavy metal ions^34^.

At the room temperature of 25 °C, the solubility product constant (*K*_*sp*_) of Cu(OH)_2_ and Zn(OH)_2_ are 5.0 × 10^–20^ and 7.1 × 10^–18^, respectively. According to the *K*_*sp*_ of metal hydroxide, the pH of metal hydroxide precipitation at different concentrations can be calculated. The calculation formula is shown in Eq. (8):

8 $$ {\text{pH}} = \frac{1}{n}lgK_{sp} - lgK_{w} - \frac{1}{n}lg\alpha_{{M^{{n^{ + } }} }} $$

where, *K*_*sp*_ and *K*_*w*_ are the solubility product constant of metal hyoxide and ion product constant of water, respectively;${\alpha }_{{M}^{{n}^{+}}}$ is the concentration of metal ions (mol/L), Where* M* represents the metal ion and *n* represents the valence of the metal ion. When the reaction temperature and the type of heavy metals are determined, according to Eq. (8), the pH that affects the precipitation of heavy metal hydroxide is only the concentration of heavy metal ions, and the higher the concentration, the lower the pH. In this study, the concentrations of Cu^2+^ and Zn^2+^ solutions used in the experiment all were 50 mg/L, according to the Eq. (8), it can be calculated that the pH of Cu(OH)_2_ and Zn(OH)_2_ precipitation are 5.90 and 6.98, respectively. The pH of BC, Cu-BC and Zn-BC are 9.33, 9.73 and 9.55, which are all higher than the pH of the precipitation of the two metal hydroxides. Therefore, precipitation is one of the mechanisms by which biochar adsorb heavy metal ions.

**Figure 8:**
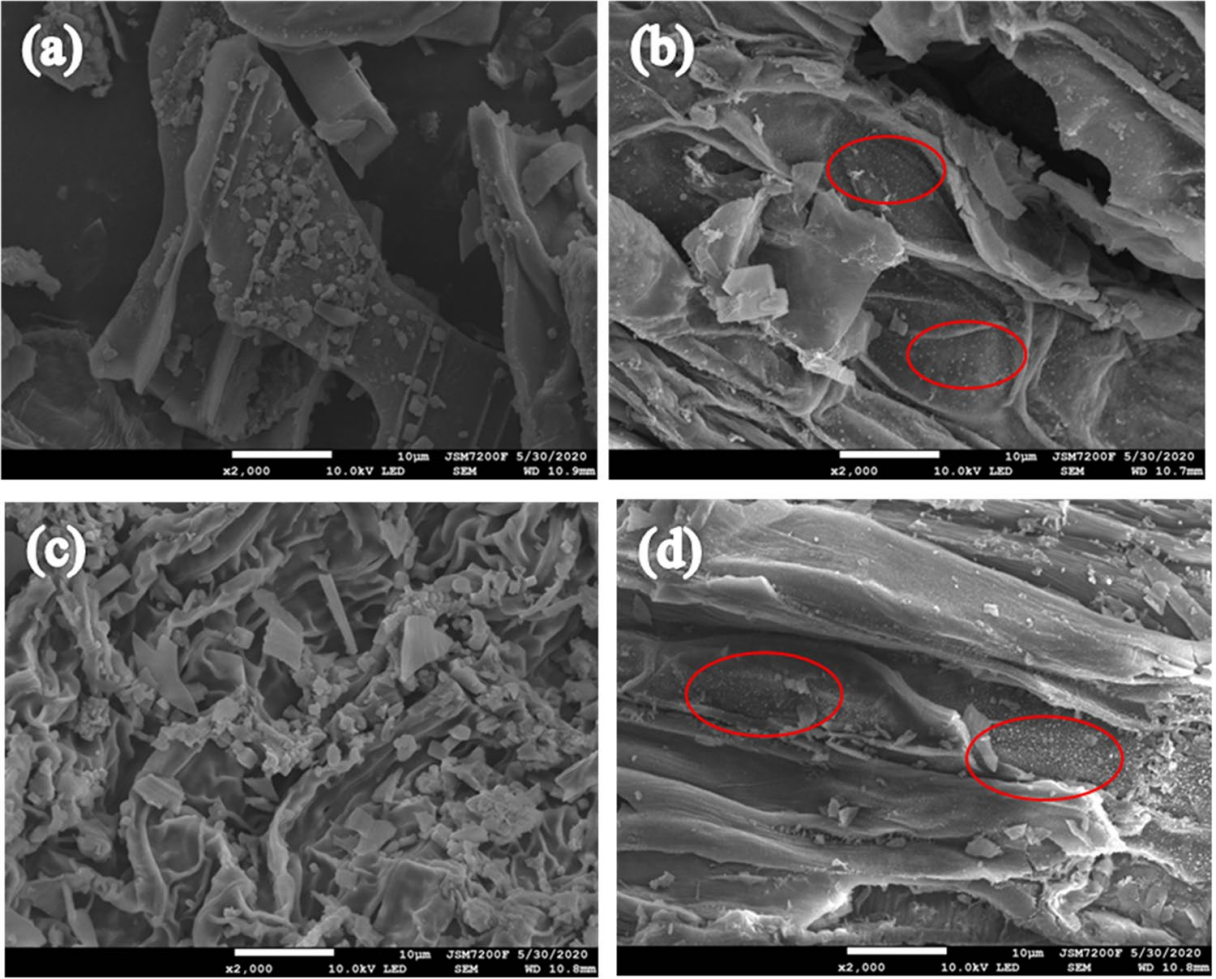
SEM images of (**a**) Cu-BC; (**b**) Cu-BC + Cu; (**c**) Zn-BC and (**d**) Zn-BC + Zn.

The SEM images of Cu-BC and Cu-BC + Cu (the Cu-BC after adsorption of Cu^2+^) were shown in Fig. 8a,b. Figure 8c,d displayed the SEM images of Zn-BC and Zn-BC + Zn (the Zn-BC after adsorption of Zn^2+^). It can be seen from the SEM images that surface of two biochars were heterogeneous, cracked, and there are a large number of fragments. These accumulated fragments make the biochar form pores, and these pores enable heavy metal ions to enter the biochar. In order to further determine the elemental composition of biochar after adsorption, the EDX analysis of biochar was carried out.

**Figure 9:**
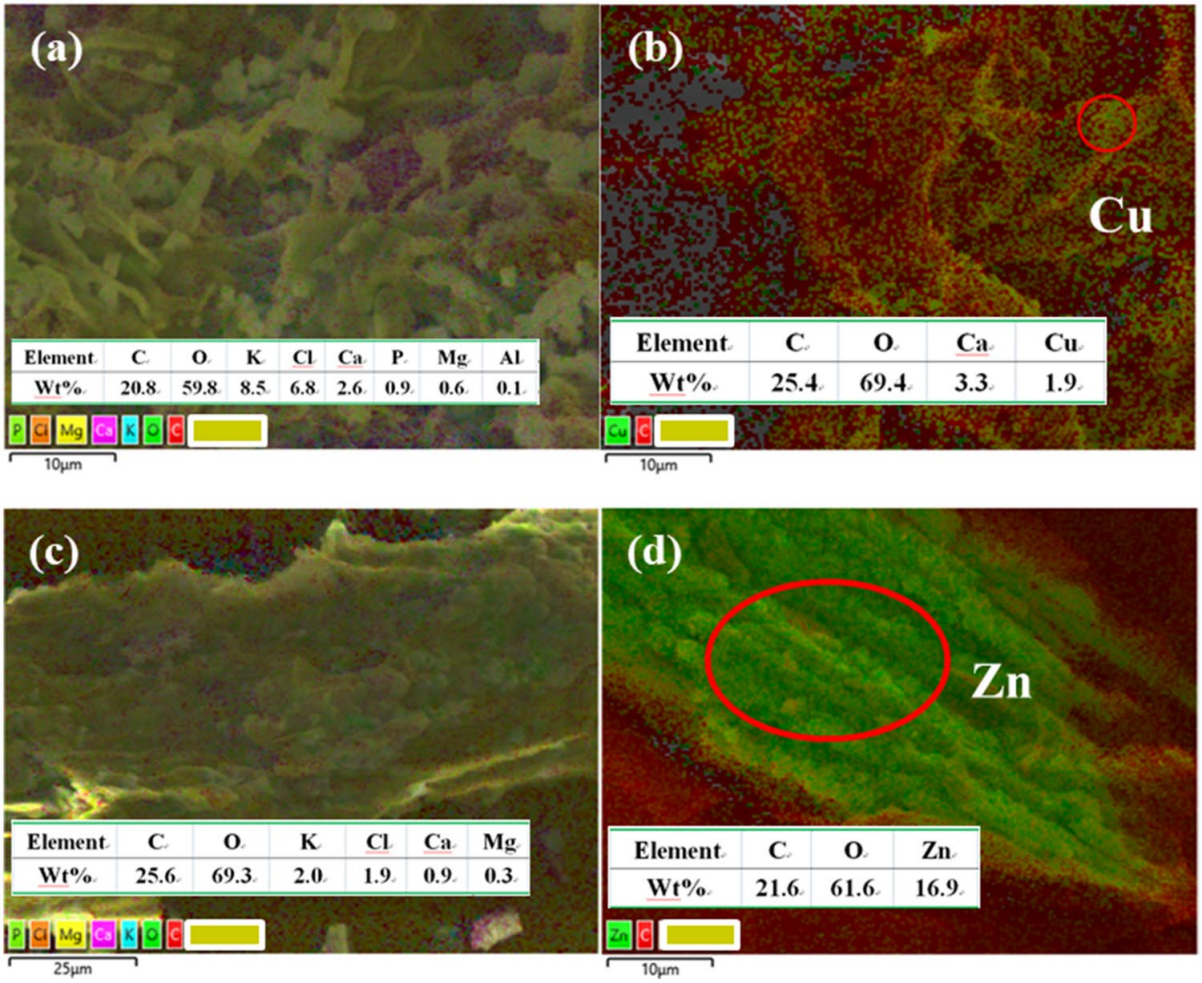
EDX layered images of (**a**) Cu-BC; (**b**) Cu-BC + Cu; (**c**) Zn-BC and (**d**) Zn-BC + Zn.

The EDX layered images of Cu-BC and Cu-BC + Cu, Zn-BC and Zn-BC + Zn are displayed in Fig. 9a–d. As can be seen from Fig. 9, Cu-BC and Zn-BC all contain K, Ca, Mg, Cl, while in the spectra of Cu-BC + Cu and Zn-BC + Zn, there are Cu and Zn elements. Studies have shown that metal ions (such as K^+^, Na^+^, Ca^2+^ and Mg^2+^) on biochar are easily attracted directly by electrostatic attraction to form metal complexes (such as –COOM and –R–O–M), and accompanied by carboxyl and hydroxyl precipitation^35^. So, these metals can be exchanged by heavy metal ions in solution by cation exchange or coprecipitation of surface complexes during the adsorption process, and the heavy metal ions were transferred from aqueous solution to biochar^36,37^.

**Figure 10:**
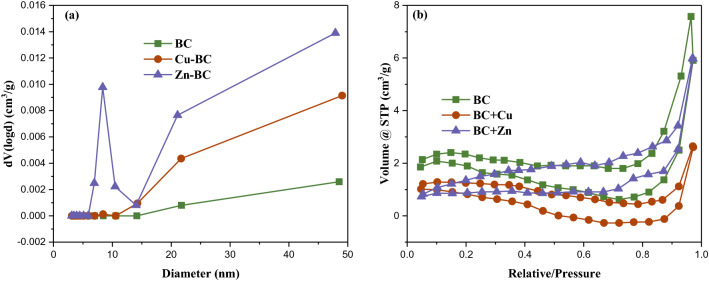
(**a**) The pore diameter distribution curves of BC, Cu-BC and Zn-BC; (**b**) N_2_ adsorption–desorption curves of BC, Cu-BC and Zn-BC.

The analysis of specific surface area pore size distribution of BC, Cu-BC and Zn-BC are presented in Table 8 and Fig. 10a, the pore diameter of BC, Cu-BC and Zn-BC are 14.505, 21.686 and 8.357 nm, which are belong to mesoporous materials and are suitable for adsorption materials^38^. But the specific surface area of Cu-BC and Zn-BC are all smaller than the BC, and this is inconsistent with the view that the specific surface area is large and the adsorption capacity is enhanced, and this also further indicates that the performance of biochar obtained by characterization means does not necessarily explain the adsorption performance of biochar. Therefore, the characterization of biochar can only be used as an auxiliary means to explain the adsorption mechanism of biochar to adsorbents, but it is insufficient to measure the adsorption performance.

**Table 8:** Parameters of specific surface area and pore structure for BC, Cu-BC and Zn-BC.

Samples	Surface area (m^2^/g)	Pore volume (cm^3^/g)	Pore diameter (nm)
BC	9.595	0.011	14.505
Cu-BC	4.299	0.005	21.686
Zn-BC	8.065	0.009	8.357

Nitrogen adsorption–desorption curves of the three adsorption materials are shown in Fig. 10b, the curves of BC, Cu-BC and Zn-BC are conformed to the IV isotherm adsorption, and type IV isotherms are derived from mesoporous adsorbent materials, with multilayer adsorption and capillary condensation phenomenon, and the hysteresis loop is H3 model, showed that the adsorption channel is relatively narrow, and the adsorption aperture for sheet pile holes^39^.

**Figure 11:**
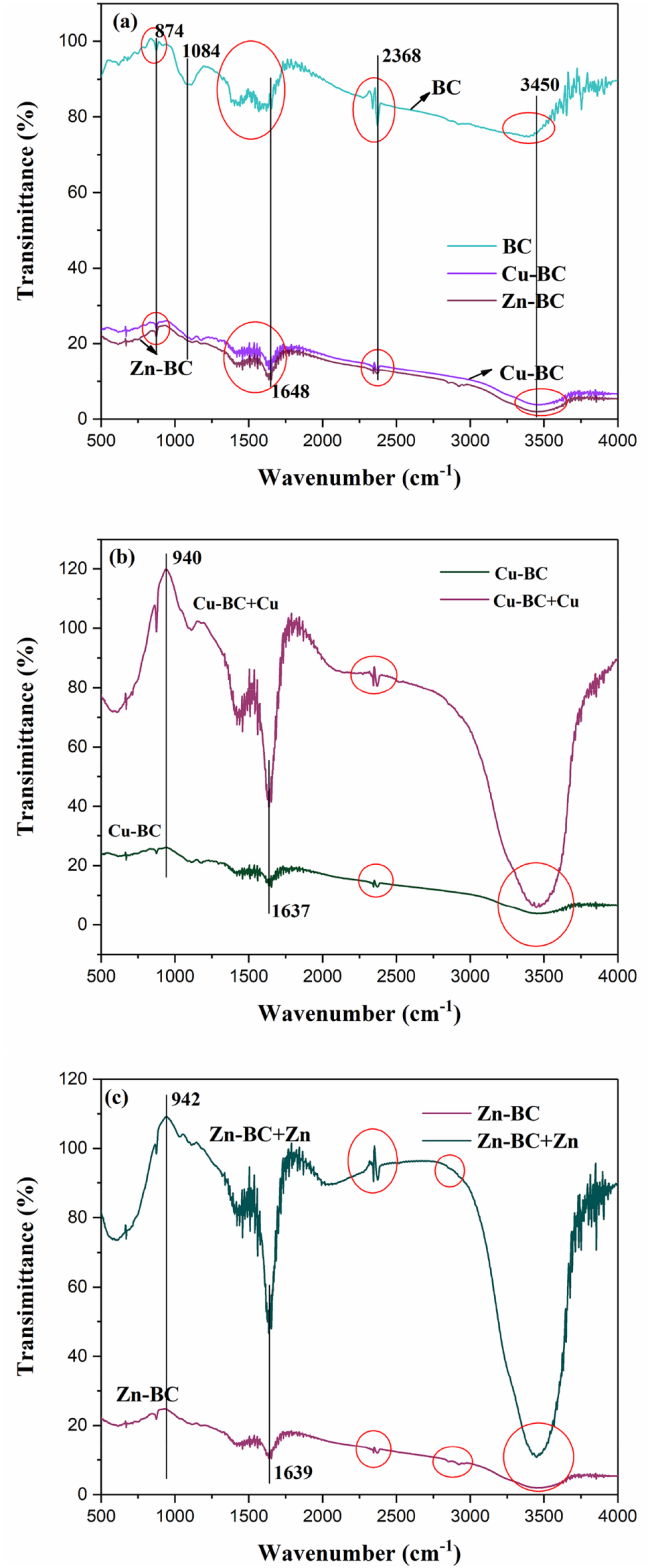
FTIR spectra of (**a**) BC, Cu-BC and Zn-BC; (**b**) Cu-BC and Cu-BC + Cu; (**c**) Zn-BC and Zn-BC + Zn.

The functional groups of biochar before and after adsorption were analyzed by FTIR, and the FTIR spectra of BC, Cu-BC and Cu-BC + Cu, Zn-BC and Zn-BC + Zn are illustrated in Fig. 11a–c. It can be seen from the Fig. 11a, the peak pattern of BC, Cu-BC and Zn-BC are almost similar, there are only some difference were noticed in the intensity and wavenumbers, which means there are only difference in the surface chemistry, and the spectra were consistent with the water hyacinth biochar which prepared by Hashem et al.^40^. Peaks around 3450/cm was observed for BC, Cu-BC and Zn-BC, attributed to the stretching of –OH, this peak exists in most biochars are prepared from water hyacinth. The transmittance of BC near 2368/cm was highest in bichars, and the intensity in the Cu-BC and Zn-BC decreased. Peaks around 1648/cm for biochars were indicated to the aromatic C=C. A peak at 1084/cm is attributed to the stretching of C–O–C stretch associated with –OH bending of cellulose, hemicellulose, and lignin. 874/cm is due to aromatic C-H out of plane bends^41–43^. The FTIR patterns after adsorption of Cu^2+^ and Zn^2+^ are displayed in Fig. 11b,c, the width and location of these peaks changed, especially at 3400 and 1500/cm, it indicates that O–H groups plays an important role in the adsorption of heavy metal ions by biochar. Compared with before adsorption, the spectra of Cu-BC + Cu and Zn-BC + Zn showed obvious peaks at 1637 and 1639/cm of the carbonyl functional group (C=O), indicating the presence of metallic carbonyl bonds^29^.

**Figure 12:**
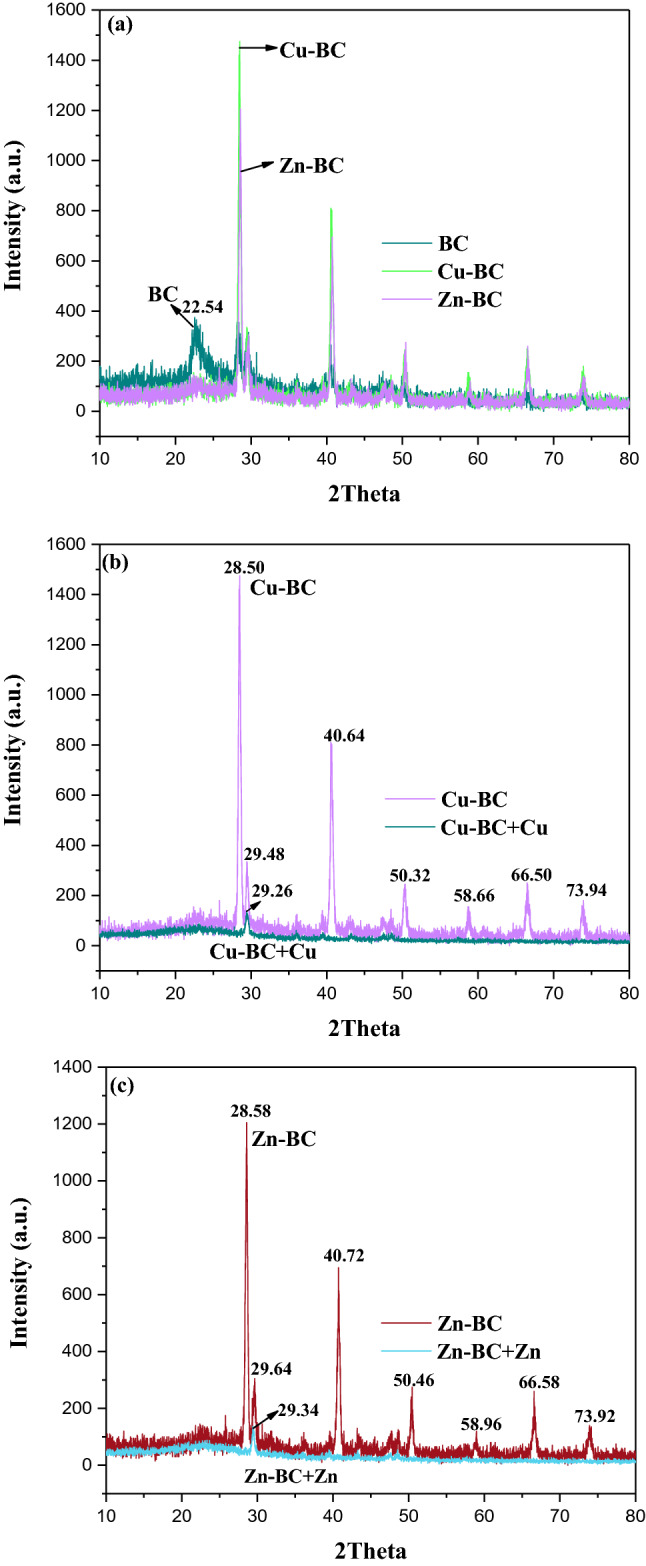
XRD spectra of (**a**) BC, Cu-BC and Zn-BC; (**b**) Cu-BC and Cu-BC + Cu; (**c**) Zn-BC and Zn-BC + Zn.

Figure 12a–c showed the X-ray diffractogram of samples. It can be seen from Fig. 12a, the spectras of Cu-BC and Zn-BC were different from BC. BC has a diffraction peak at 2θ = 22.54°, but the diffraction peak of Cu-BC and Zn-BC are not obvious here and move towards 2θ = 28.50° and 28.58°, respectively. In addition, four major peaks (2θ = 40.64°, 50.32°, 66.50° and 73.94°) were observed from Cu-BC, and for Zn-BC, four major peaks (2θ = 40.72°, 50.46°, 66.58° and 73.92°), and the four characteristic peaks of the two biochars are similar in location. Combined with the optimization results, it can be seen that the heating temperature of Cu-BC and Zn-BC are similar, which further indicates that the pyrolysis temperature has a great effect on the performance of biochar.

Figure 12b,c represented the XRD pattern of Cu-BC and Cu-BC + Cu, Zn-BC and Zn-BC + Zn, respectively. From Fig. 12a, there are more peaks were observed in the XRD results of Cu-BC and Zn-BC than in BC, which revealed that the optimized biochar contained more mineral components than the common biochar did^44^. As can be seen from Fig. 12b,c, after adsorption of Cu^2+^ and Zn^2+^, four major peaks of Cu-BC and Zn-BC all disappeared. For Cu-BC, after adsorption of Cu^2+^, the diffraction peak at 2θ = 29.48° shifts to 29.26°, and the intensity decreases, which is also the case for Zn-BC (2θ = 29.64° of Zn-BC and 29.34° of Zn-BC + Zn). These results indicate that when biochar reacts with Cu or Zn, Cu or Zn compounds are produced. Combined with EDX and FTIR analysis, Cu^2+^ or Zn^2+^ reacts with OH^−^ and CO_3_^2−^ in biochar to generate precipitate, and ion exchange reactions can also occur with cations K, Mg and Ca in biochar. It was successfully exchanged out of the water by biochar and to achieve the purpose of removal.

Through the characterization of biochar before and after adsorption, combined with the corresponding chemical basis, the adsorption mechanism of biochar for heavy metal ions mainly includes precipitation reaction, surface physical adsorption, cation exchange, electrostatic adsorption and surface complexation, etc.

## Conclusions

In this study, the effects of preparation conditions: pyrolysis time, pyrolysis temperature and heating rate on the performance of target biochar for target heavy metal ions adsorption was studied. The RSM coupled with BBD was applied to optimize the preparation parameters of target biochars. With Cu^2+^ and Zn^2+^ as the target pollutants, the production conditions of biochar adsorbed on the two heavy metal ions were optimized. The optimization results reported that two different optimal combination conditions for biochar with adsorption of Cu^2+^ and Zn^2+^. For Cu-adsorbed biochar, the optimal production conditions are: pyrolysis time of 3.09 h, pyrolysis temperature of 425 °C, and heating rate of 19.65 °C/min. While for Zn-adsorbed biochar, the optimal production conditions are: pyrolysis time of 2.19 h, pyrolysis temperature of 422 °C, and heating rate of 15.88 °C/min. The adsorption performance of Cu-BC and Zn-BC to their target pollutants is better than that of the biochar without optimized production conditions. The Langmuir isothermal model fitting the maximum theoretical adsorption capacities of BC and Cu-BC on Cu^2+^ are 177.66 and 210.56 mg/g, respectively, and the maximum theoretical adsorption capacities of BC and Zn-BC on Zn^2+^ are 146.14 and 223.32 mg/g, respectively. As general conclusion, the optimized target biochars Cu-BC and Zn-BC are effective adsorbents, which can be used for the removal of target heavy metals. This study not only prepared targeted biochar for target pollutant, but also reduced the number of experiments and the number of samples characterized to a certain extent, which has a great effect on saving raw materials, time and production costs.

## Data Availability

All data and materials are generated or used during the study appear in the submitted manuscript.
